# Effective formative assessment for pharmacy students in Thailand: lesson learns from a school of pharmacy in Thailand

**DOI:** 10.1186/s12909-023-04232-1

**Published:** 2023-05-02

**Authors:** Tida Sottiyotin, Suriyon Uitrakul, Pajaree Sakdiset, Bhudsaban Sukkarn, Thanachaporn Sangfai, Litavadee Chuaboon, Patsamon Palee

**Affiliations:** 1 School of Pharmacy, Walailak University, Thasala, Nakhon Si Thammarat, 80160 Thailand; 2 Drug and Cosmetics Excellence Center, Walailak University, Thasala, Nakhon Si Thammarat, 80161 Thailand

**Keywords:** Formative assessment, Summative assessment, Effectiveness, Pharmacy, Key success factors

## Abstract

**Introduction:**

: Formative assessment (FA) is an assessment concept that is of interest in education. The Doctor of Pharmacy program is one of the programs in which FA is usually implemented. This study aimed to describe the correlation between FA scores and summative assessment (SA) scores and to suggest possible key success factors that affect the effectiveness of FA.

**Methods:**

This study employed a retrospective design using mixed methods for data collection. Data in the semesters 1/2020 and 2/2020 of the Doctor of Pharmacy curriculum in a Thailand pharmacy school were used. Three sets of data were gathered, including the course information (e.g. FA methods, FA scores, and SA scores) from 38 records, self-reports from 326 students and 27 teachers, and 5 focus group discussions. The quantitative data were statistically analyzed using descriptive statistics and Pearson correlation, while the qualitative data were analyzed using a content analysis framework.

**Results:**

The analysis revealed five main methods that were used for FA, including individual quizzes, individual reports, individual skill assessments, group presentations, and group reports. Of all 38 courses, 29 (76.32%) had significant correlations between FA and SA scores at p-values < 0.05. The individual FA score was related to the correlation coefficient of the courses (p-value = 0.007), but the group FA score was not related (p-value = 0.081). In addition, only the frequency of individual quiz had a significant effect on the correlation coefficient. Moreover, the key success factors which affected the effectiveness of FA were divided into six themes, including the appropriate method, an effective reflection, frequency of assessment, the appropriate score, the adequate support system, and teacher knowledge management.

**Conclusion:**

The subjects that used individual FA methods provided a significant correlation between FA and SA, while those who used group FA methods did not show a significant correlation. Additionally, the key success factors in this study were appropriate assessment methods, frequency of assessment, effective feedback, appropriate scoring, and a proper support system.

## Introduction

Pharmacy courses are organized in many forms, such as lectures, laboratory, and field experience. Two types of assessments have been described for pharmaceutical education: summative and formative assessments [1]. Summative assessment (SA) makes an overall judgment of the student’s competencies. On the other hand, formative assessment (FA) reinforces students’ intrinsic motivation to learn and inspires them to set higher standards for themselves, which could improve the teaching and learning process more efficiently. Both SA and FA are designed to provide professional self-regulation and accountability [1, 2].

In general, FA is given priority at the processing level, emphasizing on conducting assessments during the learning period which may or may not be scoring. The primary aim is the assessment of learning in order to determine the current level of learners and to suggest any additional areas that teachers should develop to encourage learners to learn better. However, the results of FA are frequently reported in scoring system [3]. The Doctor of Pharmacy programs is the only pharmacy program in Thailand, which is divided into two majors: pharmaceutical care and pharmaceutical science. In order to evaluate the study performance of students in these programs, many universities in Thailand use scoring methods, such as behavior scores, quiz scores, presentation scores, and analytical thinking scores. This knowledge and ability to learn are assessed periodically throughout the course using FA, which differs from SA that assesses the competency of students only at the end of the course or in the middle and the end of the course.

Several previous studies have shown the success of FA in improving learners’ learning capability, such as the study of Baig and Gazzaz in 2020 [4]. It explored the impact of blackboard FA on the final scores in the endocrine module of third-year medical students. The results showed that the final marks were significantly higher than the scores obtained from the multiple-choice questions (MCQ), and a positive correlation was stated between online blackboard exam marks and final exam marks. The study of Cong et al. investigated the suggestions and feedback of third-year undergraduate medical students using questionnaires and also evaluated the relationship between FA and SA scores of students [5]. The students expressed their opinion that the FA not only allowed the provision of real-time feedback on the effectiveness of teaching and learning, but also nurtured self-motivation, developed analytical and problem-solving skills, and increased their collaborative efforts. The final semester scores and the proportion of students with higher scores increased after the implementation of the FA. Also, the SA scores were found to be positively related to the FA scores in the mentioned study. Another study of Yu and Li examined the effectiveness of group-based self-assessment of exam review in order to improve the comprehension of students [6]. The results revealed that students who attended the group-based FA method demonstrated an improvement of over 10% in their test scores, whereas the scores of students in the control group were improved by 2.4%. Formative assessment, which focuses on the assessment of learning for both students and teachers, is a prevailing assessment concept in the 21st century. It allows learners to identify what they already knew and what they do not yet understand, as well as plans for future learning [3, 7–10]. Although previous research revealed that FA was effective in promoting student learning and gave the benefit of improving teachers’ teaching process, these contemporary pieces of knowledge still have limitations in explaining the factors affecting the effectiveness of FA. In addition, they did not clarify the elements that make implementing the FA in the course effective.

Recently, there are two major Doctor of Pharmacy programs in Thailand which are the Doctor of Pharmacy in Pharmaceutical Care and the Doctor of Pharmacy in Pharmaceutical Science. However, some universities designed the general Doctor of Pharmacy program that does not emphasize the major area of the program, including the university of this study. Therefore, only a few subjects are different between the two majors. Regarding measurement and evaluation methods, there is no difference among those subjects in different majors. Each major still mostly uses scoring systems to evaluate the students.

This university launched a policy of implementation of FA to all subjects of the program in 2019. Since then, there has been no systematic data collection and evaluation of the FA to determine its association with the SA scores, such as mid-term or final examination scores. This study, therefore, aimed to describe the correlation between FA scores and SA scores and to suggest possible key success factors that affect the effectiveness of FA in the subjects in the Doctor of Pharmacy program of a school of pharmacy in Thailand during the academic year of 2020.

## Materials and methods

### Study design

This study focused on the subjects in the Doctor of Pharmacy program in a university during semesters one and two of the academic year 2020. This study was performed with a mixed method model; the data were collected from two main parts. First, the data that were used in evaluation of the effectiveness of FA were obtained from all subjects with more than 40 enrolled students per subject retrospectively. The data were collected using the collect information form, including course type, FA method, student individual FA scores, and student individual SA scores.

The second part was the key success factors for the effective FA. The self-report guideline was distributed to all teachers and students in the subject, which had a very high correlation between FA and SA scores. The self-report data were obtained from 27 teachers (71.05% of the total teachers in the program) and 326 students (70.71% of the students in the program). Purposive sampling was used to invite fifteen teachers and twelve students from the subjects with very high correlation to the focus group discussion (FGD) of five; four teachers and one student in a group were interviewed in-depth about key success factors of the subject. All teachers who attended FGD were course directors and/or teachers who participated in planning and evaluating each subject. While the students who participated in group discussions were information-rich informants who were course coordinators and willing to reflect on the learning process of each subject. In each teacher group discussion, the participants were asked to discuss four main questions, including “what are your methods or guidelines for designing assessments in your subjects?”, “ how do you implement formative assessments in your subject?”, “do you think each evaluation method has advantages and what are the disadvantages? why?”, and “in your opinion, what are the success factors for implementing FA in your subject?”. On the other hand, in a group of students, the discussion started with “in the subjects that you have studied, how are you assessed?” and followed with three main questions, including “which methods of assessment do you think are the most effective and least effective?”, “in your opinion, what are the success factors of FA methods which you called the most effective?”, and “do you have some suggestions for FA in the our pharmacy curriculum?”. The characteristics of the informants who participated in self-report and FGD are shown in Table 1.

**Table 1:** Characteristics of 326 students and 27 teachers who provided self-report and focus group discussion

Characteristics	Number	Percentage
Students (n = 326)		
Average age (year)	21.77 (SD = 2.34)	-
Gender		
Male	271	83.13
Female	55	16.87
Year of study		
First	46	14.11
Second	101	30.98
Third	78	23.93
Forth	61	18.71
Fifth	40	12.27
Teachers (n = 27)		
Average age (year)	39.52	-
Gender		
Male	20	74.07
Female	7	25.93
Working experience (year)		
1–5	12	44.45
6–10	10	37.03
> 10	5	18.52
Educational level		
Master degree	12	44.45
Doctor degree	15	55.55
Department		
Pharmaceutical care	13	48.15
Pharmaceutical sciences	14	51.85

### Data analysis

Formative assessment pattern was reported as mean, percentage, and standard deviation. Pearson correlation analysis was used to analyze the relationship between FA scores and SA scores. The subjects were categorized as very high correlation (r = 0.91-1.00), high correlation (r = 0.71–0.90), medium correlation (r = 0.51–0.70), low correlation (r = 0.31–0.50), very low correlation (0.00-0.30), and no correlation, based on the p-values of the correlation analysis. In addition, the relationship between the methods of FA and the correlation coefficients of every subject was analyzed with Pearson correlation analysis. Furthermore, in order to describe the optimal FA score for each type of subject to achieve a correlation coefficient, the receiver operating characteristic (ROC) curve with Youden Index was used. All statistical analyses were performed using IBM SPSS software version 27, and a p-value of < 0.05 was described as a statistical significance.

They were requested to narrate and discuss course design, FA process, problems and obstacles of FA implementation, and suggestions for FA implementation. Each focus group discussion took between 30 and 60 min. An online VDO or phone call with voice recording mode was used, and the audio record files were transcribed verbatim (word-by-word).

The FA process, the benefits of FA, problems durring FA assessments, and recommendations for FA from self-reports in each subject were collected for pooling with FGD data. All text files from self-reports and transcribed were imported into a qualitative research software, NVivo (Release 1.3) (QSR International, Victoria, Australia). Codes were created to identify participants while maintaining individual confidentiality and anonymity. An inductive thematic approach was utilized to analyze the data to reveal the key success factor in FA practice.

## Results

### FA patterns

Thirty-eight courses were included in the study, divided to 20 lecture courses (52.63%), 5 laboratory courses (13.16%), and 13 lectures with laboratory courses (34.21%). Of them, there were main five methods that were used in FA assessment, including individual quiz, individual report, individual skill assessment, group presentation, and group report (Table 2).

**Table 2:** Characteristics of formative assessment in all courses in the Doctor of Pharmacy program in a university (n = 38)

FA patterns		No. of course (percentage)	Frequency per course	Mean score (percentage) per activity
**Individual**	Quiz	32 (84.21)	12.66	2.23 ± 1.87
	Report	16 (42.11)	3.13	2.6 ± 1.38
	Skill	15 (39.47)	2.67	2.77 ± 1.72
**Group**	Presentation	31 (81.58)	1.90	7.29 ± 2.58
	Report	28 (73.68)	4.64	2.64 ± 1.85

The average frequency of all FA activities was 19.89 times per course. The used FA methods could be classified into two main groups: individual and group assessments. The 84.21% of all courses used individual quiz for assessment (12.66 times per course in average). Latter, the individual report and individual skill assessment were used in average of 3.13 and 2.67 times per course, respectively. For group assessment, 31 courses (81.58% of all subjects) used presentation, with the frequency of 1.90 times and the average score of 7.29 per course.

### The relationship between FA and SA scores

The results of 38 subjects revealed 29 courses (76.32%) in which FA and SA had a significant correlation at the p-value < 0.05. According to the Pearson correlation coefficient (r) shown in Table 3, there were 3 subjects with very high correlation, 5 subjects with high correlation, 8 subjects with medium correlation, 9 subjects with low correlation, and 4 subjects with very low correlation. After being classified by type of subject, the correlation between FA and SA was observed in 92.30% of the courses consisting of lecture and laboratory, 80% of the courses with only laboratory, and 65% of the courses with only lecture, respectively.

**Table 3:** Pearson correlation between formative assessment and summative assessment scores separated by subject (n = 38)

Subject ID	Course type	Pearson-r	p-value	Correlation
PHD-451	Lecture	0.931	0.000*	Very high correlation
PHA62-204	Lecture	0.872	0.000*	Very high correlation
PHD-331	Lecture	0.853	0.000*	Very high correlation
PHD-312	Lecture with Laboratory	0.756	0.000*	High correlation
PHD-455	Lecture with Laboratory	0.724	0.000*	High correlation
PHD-333	Lecture with Laboratory	0.662	0.000*	High correlation
PHD-532	Lecture	0.658	0.000*	High correlation
PHD-524	Lecture with Laboratory	0.655	0.000*	High correlation
PHD-454	Lecture with Laboratory	0.594	0.000*	Medium correlation
PHD-452	Lecture	0.591	0.000*	Medium correlation
PHA62-231	Laboratory	0.590	0.000*	Medium correlation
PHD-546	Lecture with Laboratory	0.572	0.007*	Medium correlation
PHD-421	Lecture with Laboratory	0.558	0.000*	Medium correlation
PHA62-205	Laboratory	0.485	0.000*	Medium correlation
PHD-422	Lecture with Laboratory	0.477	0.000*	Medium correlation
PHD-322	Lecture with Laboratory	0.468	0.000*	Medium correlation
PHD-461	Lecture	0.436	0.000*	Low correlation
PHD-341	Lecture with Laboratory	0.430	0.000*	Low correlation
PHD-525	Lecture	0.419	0.000*	Low correlation
PHA62-201	Laboratory	0.384	0.000*	Low correlation
PHD-456	Lecture with Laboratory	0.366	0.002*	Low correlation
PHD-463	Lecture	0.295	0.011*	Low correlation
PHD-311	Lecture with Laboratory	0.281	0.004*	Low correlation
PHD-321	Lecture	0.266	0.007*	Low correlation
PHA62-234	Lecture	0.251	0.010*	Low correlation
PHA62-206	Lecture	0.235	0.016*	Very low correlation
PHD-464	Lecture	0.232	0.045*	Very low correlation
PHA62-202	Lecture	0.226	0.020*	Very low correlation
PHA62-203	Laboratory	0.226	0.020*	Very low correlation
PHD-523	Lecture	0.303	0.092	No correlation
PHA62-200	Lecture	0.160	0.102	No correlation
PHA62-207	Laboratory	0.158	0.108	No correlation
PHA62-160	Lecture	0.118	0.177	No correlation
PHD-312	Lecture with Laboratory	0.106	0.280	No correlation
PHD-361	Lecture	-0.105	0.302	No correlation
PHA62-260	Lecture	-0.075	0.450	No correlation
PHA62-221	Lecture	0.040	0.689	No correlation
PHD-362	Lecture	0.015	0.884	No correlation

**Note**: very high correlation: Pearson-r = 0.91-1.00, high correlation: Pearson-r = 0.71–0.90, medium correlation: Pearson-r = 0.51–0.70, low correlation: Pearson-r = 0.31–0.50, very low correlation: Pearson-r = 0.00-0.30

Regarding the association between the frequency and score of FA methods used in the subjects and the correlation coefficient of the subjects, the results indicated that only the individual score of FA was related to the correlation coefficient of the course (r 0.431, p-value 0.007). The frequency and score of group FA were not related to the course correlation (r 0.287, p-value 0.081). Furthermore, particularly individual FA, only the frequency of individual quiz had a significant effect on the correlation coefficient of the course (Table 4). In other words, if a course had a high frequency of individual quiz, the FA score of that course was prone to relate to SA score. However, an increase in the frequency of individual report or individual skill assessments did not increase the correlation of FA and SA of the subject. Also, the proportion of FA scores for the individual quiz, individual report, and individual skill in a subject had no significant relation to FA-SA correlation.

**Table 4:** The association between the frequency and score of all methods used in formative assessment in all subjects

Variable		Course-r	P-value
**Individual**		0.431*	0.007
	Quiz		
	Frequency	0.344*	0.034
	Score	0.190	0.299
	Report		
	Frequency	− 0.041	0.807
	Score	− 0.084	0.757
	Skill		
	Frequency	0.072	0.667
	Score	0.175	0.503
**Group**		-0.287	0.081
	Presentation		
	Frequency	0.189	0.257
	Score	-0.552*	0.001
	Report		
	Frequency	0.218	0.248
	Score	-0.143	0.460

The optimal FA score for each course was analyzed using the ROC curve, and the results are shown in Table 5. For the lecture courses, the optimal individual score that achieved the minimum correlation of 0.3 was 22%. To elevate this correlation to high correlation (Pearson-r ≥ 0.7), the individual FA score of the subject should be increased to at least 25.5%. For the laboratory courses, the optimal individual score that achieved the minimum correlation of 0.3 was 20.5%. In order to achieve a higher correlation (Pearson-r ≥ 0.5), the proportion of the individual score in the subject should be at least 28.5%. Moreover, for the courses with both lecture and laboratory, the optimal individual score that achieved the minimum correlation of 0.3 was 12%, while medium or high correlation needed the proportion of the individual score of at least 27.9 or 36.0%, respectively.

**Table 5:** The appropriate individual scores of formative assessment (out of 100) for particular course type to achieve the expected minimum correlation coefficient

Course type	Minimum correlation coefficient		
	0.7	0.5	0.3
Lecture subjects	25.5	22.0	22.0
Laboratory subject	28.5	28.5	20.5
Lecture and laboratory subject	36.0	27.9	12.0

### Key success factors

Based on the results of five FGDs, the obtained key success factors could be characterized into six factors, including the appropriate method, an effective reflection, frequency of assessment, the appropriate score, the adequate supporting system, and the teacher knowledge management.

#### The appropriate method

According to the statistical analysis and data collection from the interviews, the FA methods that assessed the student individually, such as individual quiz, were more effective to reflect the learning outcomes of learners than any methods that assessed students as a group.

*“Individual quizzes were the most effective way to assess learners because it was able to reflect what they (students) understood very clearly. The knowledge that was assessed was the true thing in their own head. And as a result, it made them clearly know what they have done wrongly and why. For group assessment, we (instructors) had no idea how much each student knew because the finished work were assessed as the overall outcome of the group.” (teacher FGD, 24August2021)*

#### An effective reflection

The results showed that the feedback process was an important part of how learners recognized and accepted their learning deficiencies. Subjects that had a process to give direct feedback to the students, such as personal conversation or explanation of the answer to the test, tended to improve the learning outcome of students and provided them more opportunity to improve themselves. In addition, the feedback would be more beneficial if it was performed as two-way communication rather than only a talk of the teacher. The proper discussion made students aware of their mistakes and understand the content of the lesson better than reading the feedback on the online system or the computers.

*“When the teacher answered and discussed the exam or exercise, it made me understand the content I learned more. I knew which part that I misunderstood and which questions that I wrongly answered. This’d be much better if it was a face-to-face discussion because I could ask the teacher directly, and the teacher was able to explain it further. I didn’t like the given answers or explanation in the e-learning or e-testing (online learning system of the university).” (student assessment report, semester 1/2020)*

*“The advantage of this course was the teachers could give feedback directly to students. For instance, when students completed a quiz at the end of the topic, the teacher would give their feedbacks to the students within the following week. Luckily, the same teacher had an opportunity to teach this group of students again, so the beginning of next topic was a good time to answer quizzes and have further discussion, as well as highlight the points that should be developed to students. Personal meeting was more effective than the online written answers because some students were not interested in reading what was written.” (teacher FGD, 24 August 2021)*

#### Frequency of assessment

The results of this study indicated that the periodic assessment during teaching provided benefits for both teachers and students. However, too frequent assessments created stress, a long-term decrease in learning potential, and corrupted behavior. Therefore, appropriate times of assessment were very important.

*“I agreed that assessments should be done in regular intervals throughout the semester in order to better follow up the students and make a plan for the next topic. However, it shouldn’t be too often. The higher frequency of assessment, the greater burden on both learners and teachers, including both physical and mental burdens. I understood that it’s a human nature to fight for survival. When students were exhausted because of too much workload, but all works still needed to be done, the student’s unintended behaviors were increases. It’s a mechanism for survival. Anyway, I believed that if the system was good enough, these behaviors shouldn’t be too extreme.” (teacher FGD, 15 July 2021)*

#### The appropriate score

The inappropriate proportion of FA score resulted in a negative effect on both learners and teachers. Very high FA scores forced teachers to design more activities to assess the students to provide them higher scores. As a result, students received more assignments and it caused negative effects on time management, learning concentration, learning motivation, as well as students’ physical and mental health. Moreover, inappropriate FA score at one time, whether too much or too little, stimulated stress and anxiety among the students. Consequently, the results of the assessment could not reflect the actual learning ability of the learners.

*“Each semester, we needed to do formative assessment to collect at least 60 points (percent) of the course, so we had to create more activities or methods to convert the student learning outcomes into scores of at least 60. The easiest way to do was giving them (students) more works or more frequent exams. But the followings were more work to do, more exams and homework to give marks, and more burden for students. Exams every week resulted in stress, fatigue, and ultimately caused students didn’t want to study. Some people became physically unhealthy. This poor mental health was a long-term effect of the inappropriate score.” (teacher FGD, 30 August 2021)*

*“Lots of work, frequent exams, and works with too high scores caused lots of stress to mine because the scores decides my life. On the other hand, some subjects that had too often exams with a few points were not worth for the reading. I knew there were some (students) who didn’t care about the tests because the scores were low. So, the scores that came out couldn’t reflect how much we understand exactly because we didn’t intend to do some works. We gave up some works because we were too tired, so I’d say that the scores of some assessments were not straightforward as it should be.” (student assessment reports, semester 2/2020)*

#### The adequate supporting system

Supporting systems such as e-learning, Google form, power backup system, and internet signal were other important factors that affected the efficiency of FA practice in the course, especially online FA practice. Currently, the most used system in this university is e-learning, which is the system developed by the university. The system is fairly stable and can support a wide range of needs for teaching and learning. However, there were still problems with the e-learning system, such as the inability to allow multiple people access at the same time. There were also problems with other supporting systems, such as a power shortage that happened many times a month. These reduced the performance of students to do the FA. In addition, they caused students anxiety when their answers could not be submitted. Some students might lose their concentration during the exam due to slow internet or power outage.

*“Because of unstable system in the university such as often disconnected wifi, the system without Autosaved function made me worry during the exam. I actually had knowledge to do the exam, but when I faced the system issue which was distracting, it interfered with my exams. Sometimes, the scores I got were less than what I should have. So I thought not all exams could assess the true knowledge.” (student assessment report, semester 2/2020)*

#### The teacher knowledge management

The data from focus group discussions clearly stated that teaching and assessment processes needed to be constantly updated to accommodate changes in policies, situations, and environmental factors. Experiences in terms of problems, obstacles, or inherent management practices among the instructors were valuable. If there was a space and an opportunity to exchange and discuss these, it would make teaching management more effective.

*“The key success factors of our subject should be the teaching team because working as a team provided an opportunity to talk often about this course. If a teacher encountered a problem in the classroom or from the assessment system, he/she would share it to other teachers. So other people could modify the process for better outcomes. Indeed, the more often we talked, the more likely that teaching management was in place.” (teacher FGD, 23 July 2021)*

## Discussion

This study revealed five different FA methods implemented in the curriculum, which can be divided into two main groups: individual FA and group FA. These methods focused on different ways of the learning process. The individual assessment was a major trigger for extrinsic motivation to improve the learning outcome. On the other hand, group assessment aimed to capture the interest of the student as the tasks were usually challenging and relevant to student’s future jobs. Group assessment was closely linked to intrinsic motivation, but a good group was required to support the students until they were motivated [1, 2, 6, 11, 12].

The highlights of this study were the correlation between FA scores and SA scores of each subject. The results then showed that the individual FA score was related to the correlation coefficient of the course, but the group FA score was not related. Furthermore, particularly individual FA, only the frequency of individual quiz had a significant effect on the correlation between FA and SA. This was in agreement with the study of Carrillo-de-la-Peña et al. [13]; students who did the tests often were more likely to be successful on the final exam than those who did less frequently.

The correlation analysis of this study found that the scores of assessment were not a determining factor of correlation between FA and SA. In other words, an increase in FA score did not significantly change the relationship between FA and SA. However, too few or too many scores in each assessment might cause a negative effect on learning outcomes because of the reduction in intrinsic motivation. Weurlander et al. [11] described that FA had an effect on extrinsic and intrinsic motivation, and also affected the development of learning potential. The pressure from the individual assessments increased the time that students spent on studying, so a reward in the form of proper scores made the students try to improve their learning. However, the given scores should not be too much or too little. The scores of FA should be enough to motivate students to improve themselves in learning.

The results from student self-reports and focus group discussions supported that FA scores were less important than the feedback methods to achieve the effectiveness. To give the feedback, a two-way communication process was recommended because it made students know their mistakes and understand the content of the lesson better than writing the feedback on computer systems or online communication channels. Based on the study of Lee et al. [14], formative assessment interventions were most effective when focused on providing student-initiated formative assessment by feedback methods. The feedback should be quality, relevant to the mistakes, and allow as much duration as possible for the instructor and students. Moreover, the feedback session should be an opportunity to design more appropriate learning in the future [15–17].

The formative assessment process has been considered as an effective way to help learners develop targeted skills, standards, and outcomes if it was organized appropriately and with the right contextual conditions [18–20]. An interesting previous study revealed that teaching and learning innovations were effective in encouraging learners to improve their learning [21]. However, the application of the methods is related to the level of learning activities and the effectiveness of assessment methods because each method has a different mechanism in the development of knowledge for different level of learners. For example, flipped learning urges individual learning skills in preschool students, stimulates autonomy and critical thinking in primary school students, and improves attitudinal, mental, and interactive skills in secondary school students. Therefore, in order to explain the appropriateness of FA techniques for pharmacy students, further research may be required to identify the mechanism and effectiveness of each learning method for pharmacy students.

This study highlighted many potential factors for an effective FA, including the frequency of FA practice. The informant mentioned that a number of assessments created a great burden on both the learner and the teacher. According to the study of Cong et al. [5], more than 50% of participants had a negative impact on an increase in workload due to more often FA. The suitable supporting system is the other contextual factor that was related to the effectiveness of FA. The respondents in this study required a stable and easily accessible system, same as the participants in other studies [22–24]; the students suggested that the assessment should be able to access in any place and at any time, so the teachers must concern about the stable online facilities, especially IT technical support.

In agreement with the study of López-Belmonte et al. [25], teacher knowledge management was one of the success factors of the learning. The mentioned study highlighted the importance of teacher’s competency and teaching style to the effectiveness of teaching; although the teaching tools were powerful, they could not generate successful learning outcomes with incompetent teachers. Therefore, in addition to the self-improvement of knowledge by teachers, the course administrators should provide an opportunity for teachers to share their teaching experiences during the course.

### Limitations

This study contains several limitations that should be stated. First, this study was conducted in a school of pharmacy in Thailand. There are more than 15 schools of pharmacy in Thailand recently, so the results of this study might not be applicable to other universities, as well as other courses in the same university such as a doctor of medicine and bachelor of nursing. Second, this study was conducted with only some subjects in the curriculum of the doctor of pharmacy, which might not reflect the information of the whole program. More subjects and more students should be included in the future analysis of the program. Third, this study retrospectively collected the data by questionnaire and interview, so the possibility of recall bias should be concerned when collecting the data. Although the data were collected in a variety of ways, a possible recall bias might still affect the results. Forth, this study did not include the data from the sixth-year pharmacy students because there is no subject that they study in the university; all subjects in the sixth year are internships. Therefore, the results of this study should be implied to only year 1–5 pharmacy students.

## Conclusion

This study highlighted the high correlation between FA and SA scores in the subjects with many individual assessments rather than group assessments, regardless of the scores of each assessment. Therefore, individual assessment should be properly decided for a subject, as well as high frequency of assessment. Moreover, the key success factors of FA were suitable scores of assessment, feedback methods, supporting systems in teaching and learning, and teacher knowledge management.

## Data Availability

The datasets used and/or analysed during the current study available from the corresponding author on reasonable request.
